# Engineering *Cupriavidus necator* H16 for enhanced lithoautotrophic poly(3-hydroxybutyrate) production from CO_2_

**DOI:** 10.1186/s12934-022-01962-7

**Published:** 2022-11-05

**Authors:** Soyoung Kim, Yong Jae Jang, Gyeongtaek Gong, Sun-Mi Lee, Youngsoon Um, Kyoung Heon Kim, Ja Kyong Ko

**Affiliations:** 1 Clean Energy Research Center, Korea Institute of Science and Technology (KIST), Seoul, 02792 Republic of Korea; 2 Division of Energy and Environment Technology, KIST School, University of Science and Technology, Seoul, 02792 Republic of Korea; 3 Department of Biotechnology, Graduate School, Korea University, Seoul, 02841 Republic of Korea

**Keywords:** *Cupriavidus necator* H16, Calvin Benson Bassham (CBB) cycle, Transcriptional regulator, Carbon dioxide fixation, Lithoautotrophic culture, Polyhydroxybutyrate (PHB)

## Abstract

**Background:**

A representative hydrogen-oxidizing bacterium *Cupriavidus necator* H16 has attracted much attention as hosts to recycle carbon dioxide (CO_2_) into a biodegradable polymer, poly(R)-3-hydroxybutyrate (PHB). Although *C. necator* H16 has been used as a model PHB producer, the PHB production rate from CO_2_ is still too low for commercialization.

**Results:**

Here, we engineer the carbon fixation metabolism to improve CO_2_ utilization and increase PHB production. We explore the possibilities to enhance the lithoautotrophic cell growth and PHB production by introducing additional copies of transcriptional regulators involved in Calvin Benson Bassham (CBB) cycle. Both *cbbR* and *regA*-overexpressing strains showed the positive phenotypes for 11% increased biomass accumulation and 28% increased PHB production. The transcriptional changes of key genes involved in CO_2_—fixing metabolism and PHB production were investigated.

**Conclusions:**

The global transcriptional regulator RegA plays an important role in the regulation of carbon fixation and shows the possibility to improve autotrophic cell growth and PHB accumulation by increasing its expression level. This work represents another step forward in better understanding and improving the lithoautotrophic PHB production by *C. necator* H16.

**Supplementary Information:**

The online version contains supplementary material available at 10.1186/s12934-022-01962-7.

## Background

With increasing global CO_2_ emissions at their highest in recent years, carbon neutrality by 2050 is the most significant mission in the world to alleviate climate change. To this end, the modern industry has reformed the current production systems to minimize greenhouse gas emissions and promote the conversion of CO_2_ into value added products [1]. As industrial biotechnology has grown, the use of sustainable and industrial exhaust gas feedstocks including CO_2_, CO, and CH_4_ from various sources (e.g., steel mills, ethanol production plants, and biogases) shows to be a promising trend towards net-zero-carbon commodities [2, 3]. In order to further promote this trend, it is necessary to increase the capacity of natural organisms (e.g., plants, algae, cyanobacteria, other photo- and chemoautotrophic bacteria) for enhanced CO_2_ fixation. The development of synthetic biology has enabled promoting the microbial CO_2_ conversion into value added chemicals by engineering CO_2_-fixation pathways and energy-harvesting systems [3–5].

The Calvin-Benson-Bassham (CBB) cycle, utilizing one of the most abundant proteins on earth, the CO_2_ fixation enzyme ribulose-1,5- bisphosphate carboxylase/oxygenase (RuBisCO), is the most prevalent CO_2_ assimilation pathway widely distributed in higher plants, algae, and cyanobacteria [6, 7]. Despite its central role, RuBisCO is a notoriously inefficient enzyme that makes improving its efficiency a highly promising approach for the enhancement of CO_2_ fixation [7, 8]. There has been extensive effort on improving the catalytic activity of this key carbon fixation enzyme, but have resulted in limited success in cases of photoautotrophic cyanobacteria and algae [6, 9]. As well as improving the activity of RuBisCO itself, the CO_2_-fixation has been reinforced by regulating the expression of RuBisCO and carbon flux control enzymes (e.g., aldolase, fructose-1,6-sedoheptulose-1,7-bisphosphatase and transketolase) involved in the CBB cycle [5, 7, 10]. The regulation of cellular carbon flux can also be harnessed to reinforce CO_2_ assimilation by boosting product synthesis pathway and controlling transcriptional factors [5]. Although it is difficult to identify the effective transcriptional factor, the metabolic networks can be fine-tuned at various levels by engineering and regulating transcriptional factors. In one example, the lipid production was doubled by modulating a transcriptional factor ZnCys expression of industrial microalgae *Nannochloropsis gaditana* while retaining its autotrophic cell growth [11].

*Cupriavidus necator* H16 (formerly known as *Ralstonia eutropha*) utilizing CBB cycle for CO_2_ fixation is a hydrogen-oxidizing chemolithotrophic bacterium capable of synthesizing poly(R)-3-hydroxybutyrate (PHB), which is used as a biodegradable plastic. This bacterium can utilize CO_2_ by using hydrogen and oxygen as electron donor and acceptor, respectively [12]. *C. necator* H16 has been metabolically engineered to convert CO_2_ to PHB, ethanol, isopropanol, fatty acid, terpene, and lycopene. This suggests that the bacteria has great potential as a promising autotrophic platform strain with expanded synthetic biology tools [3, 13–18]. Assimilation of CO_2_ by *C. necator* H16 proceeds via the CBB cycle. The enzymes of the CBB cycle including CbbR, CbbL, CbbS, CbbX, CbbY, and others are encoded on two different replicons of its genome, the chromosome and mega plasmid. The polycistronic CBB expression cassette is mainly activated by the LysR-type transcriptional regulator CbbR located immediately upstream of *cbb* operon [19, 20]. Next to CbbR, the global transcriptional system RegA/RegB also plays a crucial role in the cbb promoter regulation [20]. Previously, the autotrophic cell growth and PHB production improvement have been investigated by engineering RuBisCO, carbonic anhydrase, and hydrogenases [8, 21, 22]. However, studies providing a basis for metabolic engineering of *C. necator* H16 for enhancing CO_2_ fixation efficiency are still limited.

In this work, the lithoautotrophic bacterium *C. necator* H16 was engineered to enhance the autotrophic cell growth and PHB production by overexpressing the heterologous RuBisCO. Since the control of *cbb* operon expression is influenced by the regulatory network, the transcriptional regulators *cbbR* and *regA* were also overexpressed in *C. necator* H16. The results of the transcriptional changes of genes associated with the cellular metabolism in the recombinant *C. necator* H16 were investigated. Insights gained from transcriptome analysis enhances the overall understanding of autotrophic metabolism of *C. necator* H16 and can be used for the further strain engineering in order to improve the production efficiency of PHB and other commodities from CO_2_.

## Results and discussion

### Enhanced autotrophic cell growth of engineered *C. necator* strains

**Fig. 1:**
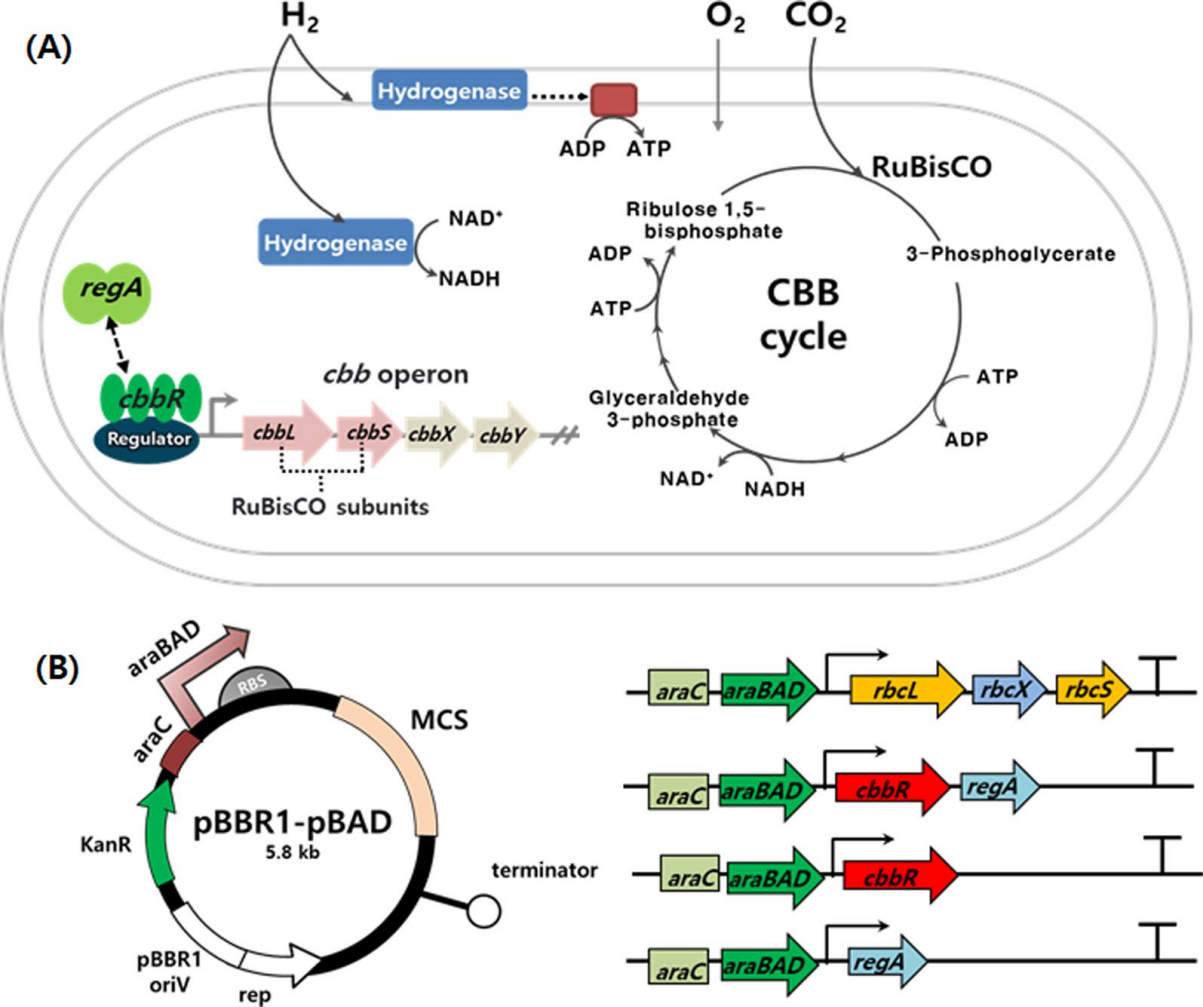
The schematic diagram of CO_2_-fixing pathway (**A**) and expression plasmids (**B**) used for the enhanced lithoautotrophic cell growth and PHB production of *C. necator* H16. Key genes (*rbcLXS* from *Synechocystis* sp. PCC6803*, regA* and *cbbR* from *C. necator* H16) involved in CO_2_-fixing metabolism were overexpressed in the lithoautotrophic bacterium *C. necator* H16. The vectors were constructed with pBBR1-MCS2 bearing L-arabinose inducible araBAD promoter (PBAD)

The CBB cycle is often limited by its low catalytic rate and some of its enzymes are more critical than others towards enhancing the efficiency of CO_2_ fixation. In this study, the key enzyme of CBB cycle enzymes, ribulose-1,5-bisphosphate carboxylase/oxygenase (RuBisCO) and transcriptional regulatory proteins (CbbR and RegA) were selected to be overexpressed in *C. necator* H16 to improve the lithoautotrophic cell growth. Overexpression plasmids with the heterologous RuBisCO genes (*rbcL, rbcS* and *rbcX*) derived from *Synechocystis* sp. PCC6803 or the native regulatory genes including *cbbR* and *regA* were constructed based on the pBBR1-MCS2 multiple-copy vector as listed in Fig. 1 and Table 1. The recombinant strains were subjected for autotrophic gas fermentation in minimal medium supplemented with the gas mixture (O_2_:H_2_:CO_2_ = 10:80:10).

**Table 1:** Strains and plasmids used in this work

Strains	Description	Source
*C. necator* H16	Wild type gentamycin resistant (Gen^r^)	ACTC 17699, KCTC 22469
CbbR	*C. necator* H16 carrying pCbbR	This study
RegA	*C. necator* H16 carrying pRegA	This study
CbbR-RegA	*C. necator* H16 carrying pCbbR-RegA	This study
RbcLXS	*C. necator* H16 carrying pRbcLXS	This study
Plasmids		
pBBR1MCS2	Broad host range plasmid; P_Lac_, Km^r^	Addgene
pBAD	pBBR1MCS2-derived expression vector containing L-arabinose inducible araBAD promoter and araC; Km^r^	This study
pCbbR	pBAD with *cbbR*; Km^r^	This study
pRegA	pBAD with *regA*; Km^r^	
pCbbR-RegA	pBAD with *cbbR*-RBS-*regA*; Km^r^	This study
pRbcLXS	pBAD with *rbcL*-RBS-*rbcX*-RBS-*rbcS*; Km^r^	This study

**Fig. 2:**
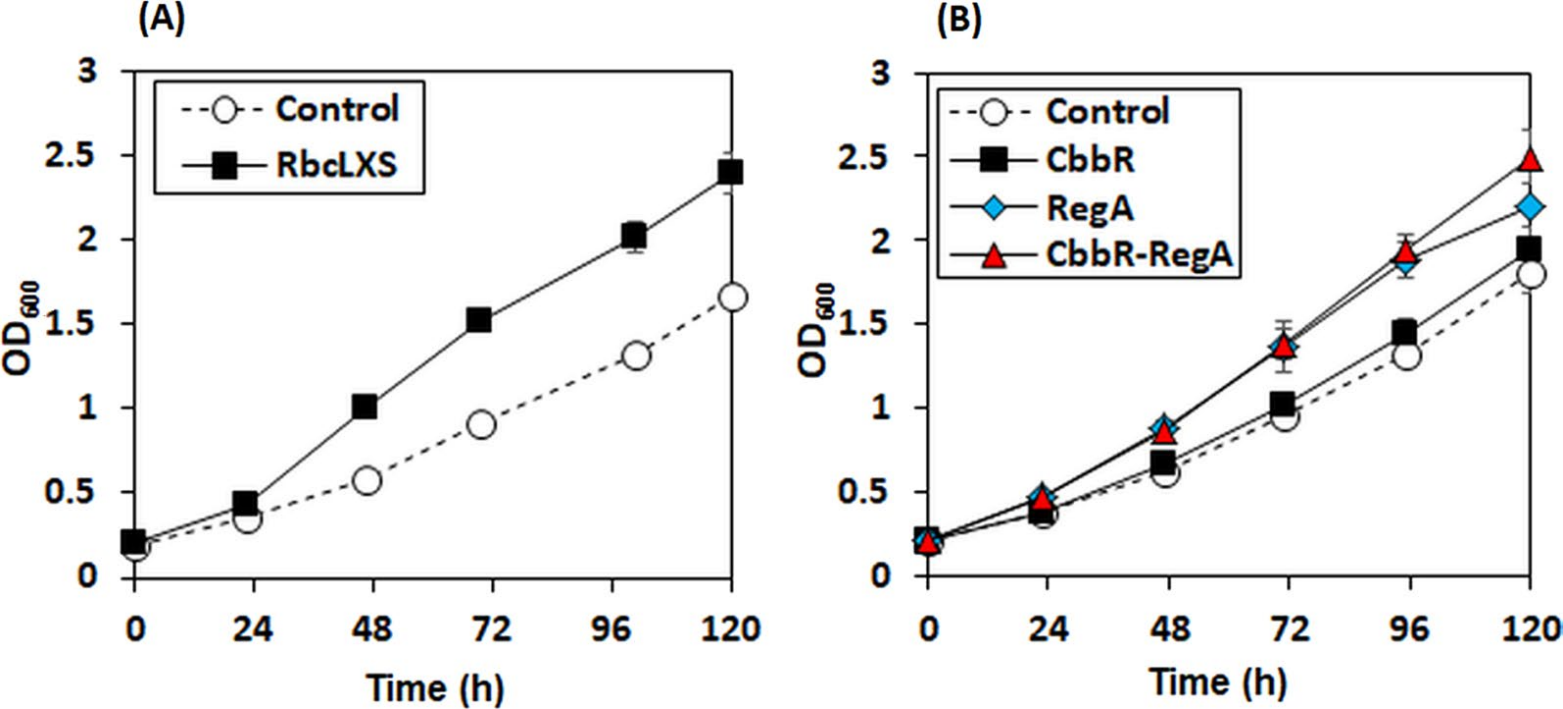
Lithoautotrophic cell growth curves of the engineered strains overexpressing **A** the heterologous RuBisCo (*rbcLXS*) and **B** the transcriptional regulators (*cbbR* and/or *regA*) under nitrogen-rich conditions (1 g/L (NH_4_)_2_SO_4_). The gene of interest was induced by adding 0.2% (w/v) of l-arabinose after 24 h of culture. The *C. necator* H16 strain harboring pBAD empty vector was used as a control. The data represent the means of triplicate experiments

As the ultimate limiting step in the CBB cycle, RuBisCO catalyzes the carboxylation reaction of ribulose-1,5-diphosphate (RuBP) and CO_2_ to generate 3-phosphoglycerate (3-PGA) for fixing CO_2_ into organic compounds, but its catalytic rate and efficiency are low [5]. Despite its abundancy in nature, the evolution of RuBisCO into a more efficient enzyme has been constrained due to the trade-off between CO_2_ affinity and carboxylation rate [7, 23]. Unlike most RuBisCOs, the *C. necator* RubisCO seems to be evolved to retain optimal carboxylation rate in aerobic conditions with abundant competing O_2_ [24]. However, its catalytic activity of carboxylation is known to be lower than those of cyanobacterial RuBisCOs [25]. We therefore intended to improve the autotrophic cell growth of *C. necator* H16 by overexpressing alternative RuBisCO enzymes with more favorable kinetic parameters at first. Among the cyanobacterial RuBisCOs exhibiting high catalytic carboxylation activities, genes derived from *Synechocystis* sp. PCC6803, a well-studied and widely used model cyanobacterium, were chosen to be overexpressed. When RuBisCO genes (*rbcL* and *rbcS*) derived from *Synechocystis* sp. PCC6803 were overexpressed with the assembling chaperone gene *rbcX*, a higher cell growth rate was maintained compared to the control throughout 120 h (Fig. 2A). This is in line with previously reported findings that show the heterologous expression of *rbcL* and *rbcS* enhances the production of enzymatically active RuBisCO upon coexpression with *rbcX* [26]. In *Synechocystis* sp. PCC6803, RubisCO is encoded by an operon in the order *of rbcL-rbcX-rbcS*. RuBisCO folding and assembly are complex processes involving chaperons that vary between species, thus making the heterologous RuBisCO expression strategy highly restricted [27, 28]. This findings of this study indicated that the assembly of a functional cyanobacterial RuBisCO in *C. necator* was successfully achieved with the aid of cyanobacterial *rbcX*. However, the overexpression of endogenous *cbbLS* with *groES/EL* chaperonin did not enhance the autotrophic cell growth (data not shown). Li et al. [8] also showed the feasibility of improving autotrophic cell growth rate of *C. necator* by overexpressing the heterologous RuBisCO enzyme derived from *S.* PCC7002 with assistance of endogenous GroES/EL chaperones although no significant enhancement of cell growth was observed after 96 h. Previous studies have shown that heterologous or hybrid overexpression of RuBisCOs to be positive for the enhanced carbon assimilation in many microorganisms, which include photosynthetic bacteria [6, 29–31], lithoautotrophic bacteria (e.g., *Cupriavidus necator*) [8], *E. coli* [32], and higher plants (e.g., tobacco) [33, 34]. Since RuBisCOs from cyanobacteria tend to have a faster carboxylation rate compared to other sources, many experiments have been conducted to improve photosynthesis of higher plants and bacteria by overexpressing cyanobacterial RuBisCOs despite its complex regulatory system. This makes the homologous/heterologous expression challenging [29].

While improving carbon fixation has mostly focused on enhancing the CO_2_ fixing enzyme RuBisCO, another promising strategy that was employed was overexpressing the transcriptional regulators including *cbbR* and/or *regA* (Fig. 2B). However, the overexpression of *cbbR*, the master regulator of the *cbb* operons, did not confer the significant improvement of autotrophic cell growth. Due to the heavy energy demands on the cell during CO_2_ assimilation, the expression levels of *cbb* gene clusters may be up- or down-regulated depending on the carbon-state of the cell [20, 35, 36]. While the overexpression of *cbbR* caused a minor improvement in cell growth, both *regA* only and *cbbR/regA* overexpressions significantly benefited the autotrophic cell growth of *C. necator*. These data demonstrated that RegA plays an important role in the regulation of carbon fixation and shows the possibility to increase autotrophic cell growth and biomass accumulation by increasing the expression level of RegA.

### Increased PHB production by co-overexpressing the transcriptional regulators RegA and CbbR

**Fig. 3:**
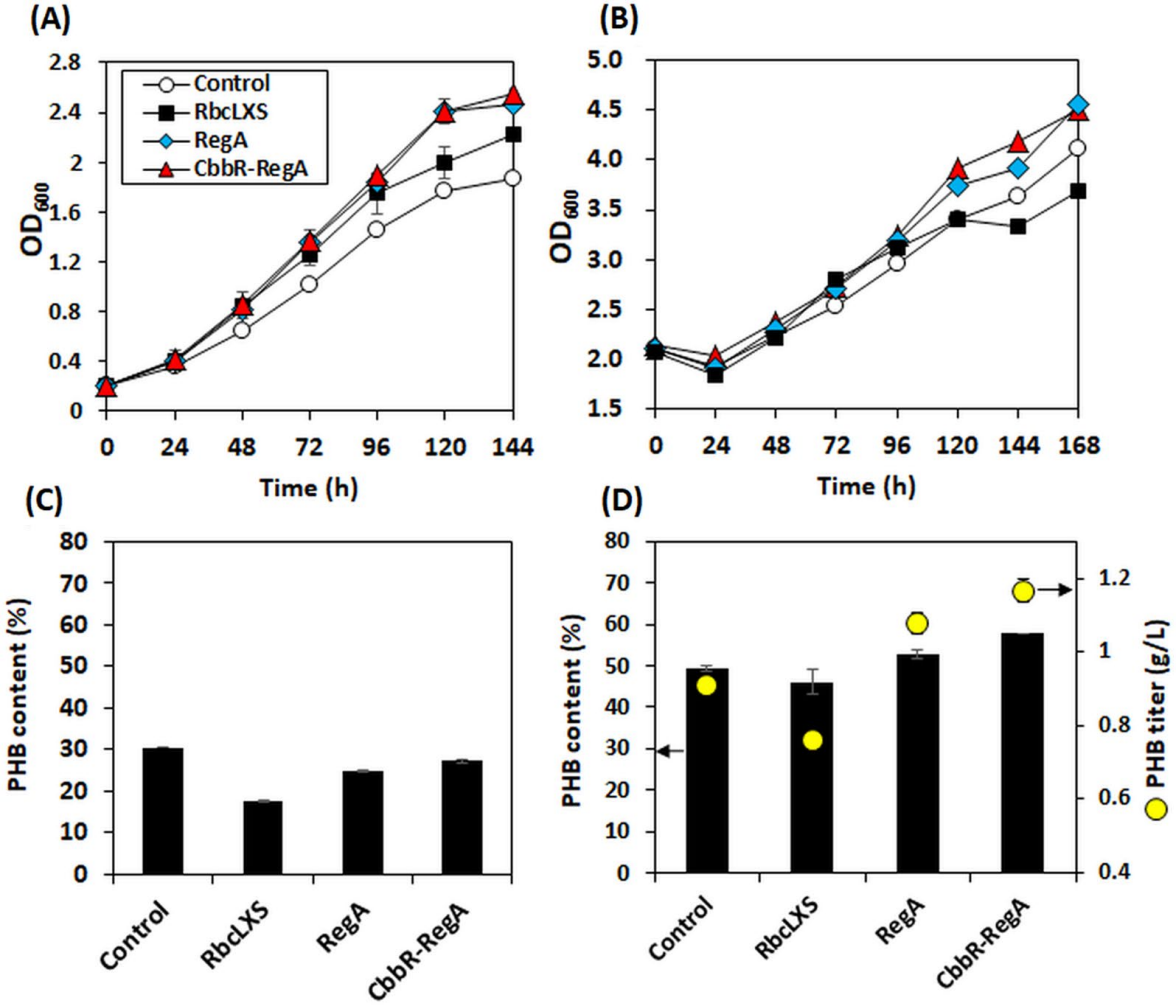
Lithoautotrophic cell growth curves of the engineered strains overexpressing the heterologous RuBisCo (*rbcLXS*) and the transcriptional regulators (*cbbR* and/or *regA*) under nitrogen-limited conditions (0.2 g/L of (NH_4_)_2_SO_4_) with the different initial optical densities of 0.2 (**A**, **C**) and 2 (**B**, **D**), respectively. The gene of interest was induced by adding 0.2% (w/v) of L-arabinose after 24 h of culture. The *C. necator* H16 strain harboring pBAD empty vector was used as a control. The data represent the means of triplicate experiments

The above studies demonstrated that overexpressing the cyanobacterial RuBisCO or transcriptional regulator *regA* resulted in the increased autotrophic cell growth. To further investigate their potential, the engineered strains were cultured under nitrogen-limited condition, allowing the question whether the increased CO_2_ fixation resulting from RuBisCO or *regA* overexpression can be utilized to enhance the accumulation of target product PHB to be addressed (Fig. 3). The PHB content of the *cbbR/regA* overexpressed strain increased from 6.3 (at 24 h) to 27.3% (at 168 h) when the *cbbR/regA* was induced by adding arabinose at 24 h (Additional file 1: Fig. S1). Compared to the control, the *cbbR/regA* and *regA* overexpressed strains exhibited comparable cell growth, but this was not correlated to the improved PHB production when the initial OD_600_ was 0.2 (Fig. 3A and B). Moreover, the PHB content of *rbcLSX*-overexpressing strain decreased by 42%. The level of nitrogen limitation may not have been optimal for boosting PHB accumulation at the initial OD_600_ of 0.2. For this reason, a higher initial cell density (OD_600_ of 2) was used in the following autotrophic fermentation experiments which allowed nitrogen deprivation to be accelerated. As shown in Fig. 3B and D, both *regA* and *cbbR/regA*-overexpressing strains with the initial OD_600_ of 2 showed the positive phenotypes for biomass accumulation as well as PHB production. Cells grown under this condition showed about 11% and 28% increased biomass accumulation and PHB titer, respectively. The PHB content (% of dry cell weight) also increased from 49 to 58%. The enhanced PHB production might be resulted from the re-distribution of carbon flux towards PHB accumulation rather than cell growth under nitrogen-deficient conditions.

However, this phenomenon did not appear to be true for *rbcLXS* recombinant strain. The strain with the heterologous RuBisCO overexpression was found to produce 16% less PHB than the control strain, implying that an increase in cell growth did not lead to a corresponding increase in carbon-based product formation. When the carbon fixation was enhanced by improving RuBisCO, the strain might channel more carbon fluxes towards cell growth, instead of towards PHB production. This result was in contrast to the previous studies that showed faster cell growth with increased production of target chemicals in many photosynthetic cyanobacteria overexpressing RuBisCO. For example, the increased isobutyraldehyde and fatty acid in cyanobacterial hosts with additional RuBisCO genes were reported [37, 38]. Regarding PHB production using *C. necator* H16, overexpressing *regA* showed to benefit both autotrophic cell growth and PHB production in the present study. Along with CbbR, the transcription of *cbb* operons additionally involves global transcription regulation system composed of RegA and RegB [20]. While the *cbb* operon transcription regulation by CbbR is influenced by the carbon state of the cell (e.g., phosphoenolpyruvate (PEP)), RegA interacts with CbbR and finely tunes transcriptional control scenario in response to the redox state of the cell [20]. The RegA/RegB system has an important role in the transcription of proteins involved in the control of energy-utilizing and energy-generating processes such as carbon fixation, nitrogen fixation, hydrogen utilization, respiration, electron transport and denitrification [20]. The RegA/RegB two-component system imposes additional layers of redox control over the energetically costly process of CO_2_ assimilation and its involvement in the *cbbR* and *cbb* operon control is well identified in *Rhodobacter capsulatus* and *R. sphaeroides* [39, 40]. To further investigate the effect of *cbbR* and *regA* co-overexpression on the autotrophic cell metabolism of *C. necator* H16, a global transcriptional analysis was performed in the next section.

### Global transcriptional profiling of cbbR/*regA* overexpressed *C. necator* H16

Genome-wide transcriptional analysis was performed using RNA sequencing to identify genes that are differentially expressed in response to the transcriptional regulatory gene (*cbbR* and *regA*) overexpression. We analyzed transcripts of the mid-exponential phase of the autotrophically grown cells under nitrogen-limited condition with the initial OD of 2. A total of 951 genes exhibited > twofold changes in expression (*p* < 0.05) in the engineered strain when compared to those of the control strain. Of these 951 genes, roughly half were observed to be upregulated.

**Fig. 4:**
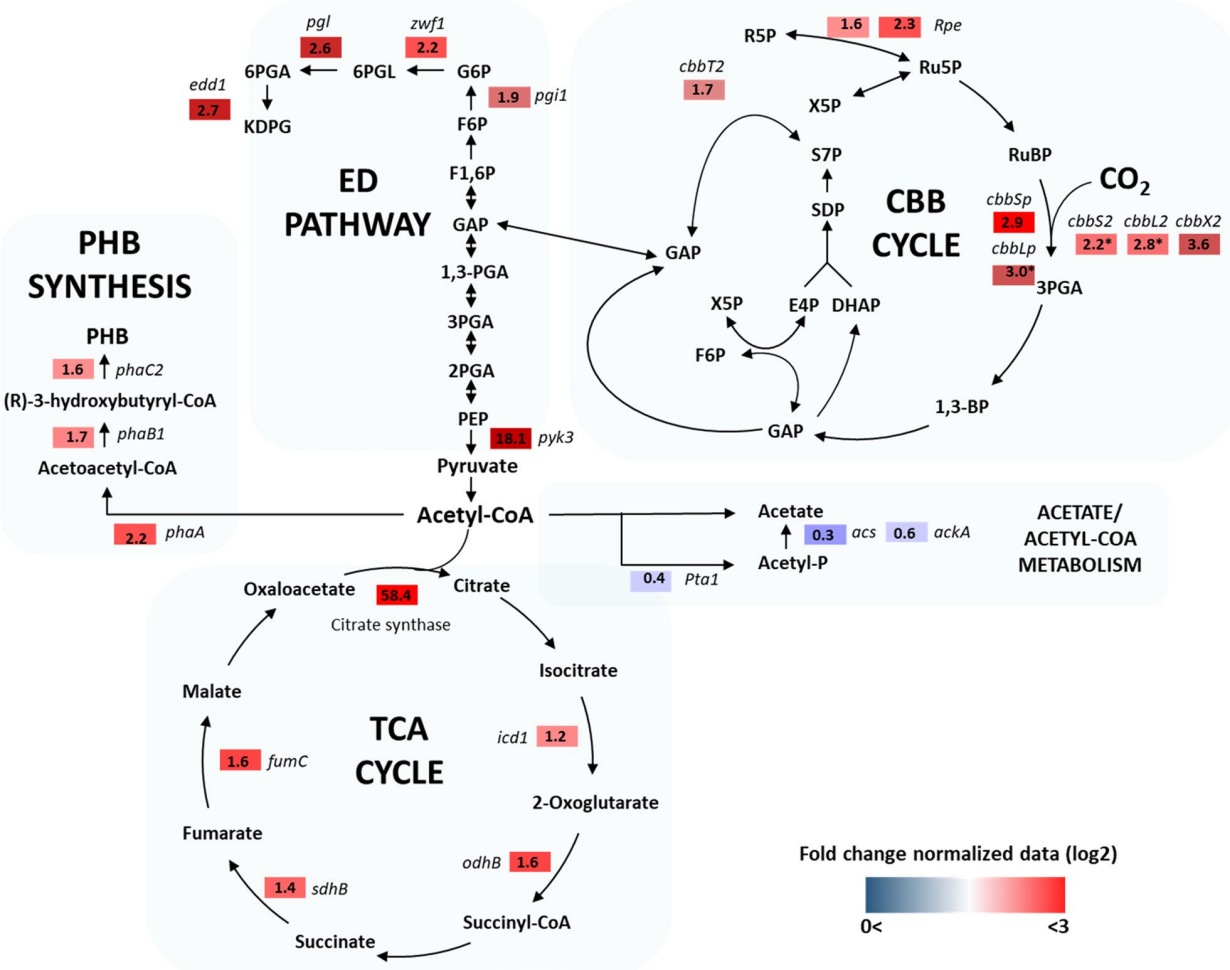
Global transcriptomic changes of genes involved in the major carbon metabolism in *C. necator* H16. In transcriptomic comparative analysis, the cells overexpressing both *cbbR* and *regA* (CbbR-RegA strain) was the experimental group while the strain harboring the empty vector was the control group (Fold changes > 1.5, p value < 0.05)

Table 2 and Fig. 4 show the differentially expressed genes categorized according to CBB, tricarboxylic acid (TCA), and Entner-Doudoroff (ED) pathways. Since *regA* and *cbbR* were overexpressed in the engineered strain, those genes were significantly upregulated. Although *regB* as the component of regulatory system *regA*/*regB* was not overexpressed, a 4.3-fold increase of its expression level was also detected. Since RegB, a membrane associated histidine sensor kinase, phosphorylates its cognate response regulator RegA to stimulate the binding of CbbR to the *cbb* promoter region to regulate the transcription of *cbb* operon, its expression might be enhanced as well [20, 41]. By overexpressing the regulatory proteins, genes involved in CBB and TCA cycle were observed to be generally up-regulated in the engineered strain. Most of the enzymes required for the CBB cycle are encoded in the *cbb* operon, present in both the chromosomal and plasmid-borne clusters in *C. necator* H16 while the regulatory *cbbR* gene forms a monocistronic operon within the chromosomal *cbb* cluster [40]. As shown in Table 1, large and small subunits of RuBisCO (*cbbL* and *cbbS*) of the chromosomal and plasmid-borne clusters were > twofold up-regulated while the RuBisCO accessory protein *cbbX* and *cbbY* located immediately downstream of the chromosomal RuBisCO gene *cbbLS* increased by 3.6-fold. The upregulated genes involved in the CBB cycle might be affected by RegA/RegB in combination with CbbR which play a crucial role in the transcriptional controls of both chromosomal and plasmid-borne *cbb* promoters. Among the TCA cycle related genes, citrate synthase encoding genes were highly up-regulated by 22–58.3 folds. Pyruvate kinase gene, pyk3, catalyzing the conversion of phosphoenolpyruvate (PEP) to pyruvate with the production of ATP was also upregulated 18-fold.

**Table 2:** Transcriptional changes of genes involved in carbon metabolism of *cbbR* and *regA*-overexpressing strain

Metabolism	Gene	Description	Fold change
Regulatory protein	*regA* (H16_A0202)	Response regulator containing a Fis-type DNA-binding domain two-component system	266
	*regB (H16_A0203)*	HAMP domain-containing histidine kinase	4.3
	*cbbR* (H16_B1396)	LysR family transcriptional regulator, low CO_2_-responsive transcriptional regulator	25628
CBB cycle	*cbbS2*	RubisCo small subunit (encoded on chromosome2)	2.2*
	*cbbL2*	RubisCo large subunit (encoded on chromosome2)	2.8*
	*cbbSp* (PHG426)	RubisCo small subunit (encoded on megaplasmid)	2.9
	*cbbLp* (PHG427)	RubisCo large subunit (encoded on megaplasmid)	3.0*
	*cbbX2* (H16_B1393)	Rubisco accessory protein CbbX, AAA ATPase	3.6
	*cbbY2* (H16_B1392)	HAD family hydrolase, unknown function	2.7
	*cbbE2* (H16_B1391)	ribulose-phosphate 3-epimerase; rpe	1.6*
	*cbbEp* (PHG423)	ribulose-phosphate 3-epimerase; rpe	2.3*
	*cbbTp (PHG420)*	Transketolase; tkt	1.3
	*cbbT2 (H16_B1388)*	Transketolase; tkt	1.7
TCA cycle	H16_B0357	Citrate synthase	58.3
	H16_B0414	Citrate synthase	22
ED pathway	*pyk3* (H16_B0961)	Pyruvate kinase, the conversion of phosphoenolpyruvate and ADP to pyruvate and ATP	18.0
	*glk* (H16_B2564)	Glucokinase	3.4
	H16_A1178	Phosphogluconate dehydratase	2.7
	H16_B1213	2-Keto-3-deoxy-6-phosphogluconate aldolase	1.8
	*zwf1* (H16_A0316)	Glucose-6-phosphate 1-dehydrogenase, NADPH generation	2.2
	*pgl (H16_B2565)*	6-Phosphogluconolactonase	2.6
	*edd1*	phosphogluconate dehydratase	2.7

Fold changes > 1.5, p value < 0.05

*p value < 0.1

Since pyruvate and acetyl-CoA are key to the PHB synthesis from carbon dioxide in *C. necator* H16, genes related to those metabolisms were therefore analyzed. As shown in Fig. 2 and Table 3, the main PHB production gene cluster *phaCAB* except *phaC1* did appear to exhibit changes in their expression levels. Although the expression level of *phaC2* was 1.6-fold up-regulated, it might not directly affect the PHB synthesis since it has no activity. In particular, ß-ketothiolase (*phaA1*) involved in the first step of PHB synthesis condensing two moles of acetyl-CoA to acetoacetyl-CoA, was 2.2-fold up-regulated. Furthermore, phosphate acetyltransferase encoding genes converting acetyl-CoA to acetate such as *pta1* (H16_B1631), *ackA* (H16_B1630) and *ackA2* (H16_A0670) were observed to be down-regulated. These findings indicate that there is a change in the flux of acetyl-CoA in the cell, redirecting the acetyl-CoA into the cell growth and PHB synthesis, since the precursor of PHB is produced using two molecules of acetyl-CoA. A number of studies have revealed that the PHB production is highly dependent on the intracellular availability of acetyl-CoA [42–44]. Also, PHB biosynthesis in *C. necator* H16 could possibly be enhanced through the up-regulation of NADPH generation-related *zwf* gene encoding glucose-6-phosphate dehydrogenase (G6PDH) [45].

**Table 3:** Transcriptional changes of genes involved in the metabolism of acetyl CoA and PHB synthesis in *cbbR* and *regA*-overexpressing strain

Metabolism	Gene	Description	Fold change
Acetyl-CoA related metabolism	*acs* (H16_A1616)	Acetyl-coA synthetase; ATP + Acetate + CoA ↔ AMP + Diphosphate + Acetyl-CoA	0.34
	*pta1* (H16_B1631)	phosphate acetyltransferase; acetyl-CoA → acetate	0.44
	H16_B1102	acetyl-CoA synthetase; ATP + Acetate + CoA ↔ AMP + Diphosphate + Acetyl-CoA	0.28
	*ackA* (H16_B1630)	Phosphate acetyltransferase-acetate kinase pathway, acetyl-CoA → acetate	0.63
	*ackA2* (H16_A0670)	Phosphate acetyltransferase-acetate kinase pathway, acetyl-CoA → acetate	0.65
PHB synthesis	*phaA* (H16_A1438)	acetyl-CoA acetyltransferase	2.2
	*phaB1* (H16_A1439)	acetoacetyl-CoA reductase	1.7
	*phaC2* (H16_A2003)	polymerase subunit PhaC	1.6
	*phaZ1* (H16_A1150	PHB depolymerase	0.27
	*phaZ2* (H16_A2862)	PHB depolymerase	2.0

Fold changes > 1.5, p value < 0.05

The expression level of carbonic anhydrase, *can*, which catalyzes the interconversion between carbon dioxide and bicarbonate (CO_2_ + H_2_O ↔ HCO_3_^−^ + H^+^) was found to be increased 1.8-fold (Additional file 1: Table S1). In a previous study, the *can* gene-overexpressed *C. necator* strain revealed a 1.5-fold increase in PHB accumulation [22]. While cyanobacteria use carboxysome as a unique CO_2_ concentrating mechanism to enhance its fixation efficiency, *C. necator* lacking this system expresses four carbonic anhydrases [21, 22]. In addition to the key CBB cycle enzymes, carbonic anhydrase is of great importance to maximize CO_2_ concentration near RuBisCO in autotrophic metabolism [21]. Since *C. necator* H16 is able to fix CO_2_ through CBB cycle using hydrogen as the energy source, the transcriptional changes of hydrogenases were also investigated. However, the expression levels of genes encoding membrane-bound [NiFe] hydrogenases (*hoxG* and *hoxK*) and soluble hydrogenases (*hoxF, hoxU, hoxY*, and *hoxI*) except *hoxH* (2.5-fold down-regulated), were not significantly changed.

The transcription levels of majority genes encoding flagella were also down-regulated (Additional file 1: Table S1). The gene *fliC*, the main structural protein of bacterial flagella, was downregulated 5 folds and the changes in flagellations at each growth phase have been reported. Flagellation of cells is stagnated in the stationary phase allowing PHB accumulation while cells appear to be strongly flagellated in the early exponential phase [46]. In the present study, the down-regulation of flagella gene clusters in the engineered strain may contribute to enhancing the PHB accumulation. Previous studies have shown that disrupting gene clusters relevant to outer membrane including flagella and pili benefited the PHB accumulation [47, 48]. Since the biosynthesis and assembly of various flagella and pili components requires high energetic cost, their biosynthesis might create a substantial metabolic burden [48]. The decrease in flagella biosynthesis could help in saving energy and improve autotrophic cell growth and PHB accumulation in *C. necator* H16. Disrupting gene clusters relevant to flagella or pili may be an efficient strategy to improve cell performance to accumulate PHB. Altogether, these findings provide a reference for the construction of metabolic engineering *C. necator* H16 towards high efficiency for PHB production under lithoautotrophic cultivation conditions.

## Conclusion

In this study, the overexpression of *cbbR* and *regA* in *C. necator* H16 enabled the improvement in its autotrophic cell growth and PHB accumulation. The global transcriptional regulator RegA seems to play an important role in the regulation of carbon fixation and shows possibility of enhancing the industrial applicability of *C. necator* H16. The comparative transcriptome provides references for the enhancement of PHB synthesis using carbon dioxide under autotrophic conditions. In summary, this study represents another step forward to better understanding the lithoautotrophic PHB production by *C. necator* H16.

## Methods

### Strains and plasmids

*Cupriavidus necator* H16 (KCTC 22469; Korean Collection for Type Cultures, Daejeon, Korea) was used in this study. All strains used in this study are listed in Table 1.

### Culture conditions

*Cupriavidus necator* H16 was routinely cultivated at 30 °C and 200 rpm. To grow *C. necator* strains autotrophically, the glycerol stock was first inoculated in a rich Luria–Bertani (LB) broth for 24 h and then pre-cultured in a 250 ml flask containing a minimal media with 10 g/L fructose for 24 h. Cultures were centrifuged at 4,200 rpm for 10 min, and the harvested cell was washed twice with the minimal medium. The final composition of the minimal medium was 6.74 g/L Na_2_HPO_4_·7H_2_O, 1.5 g/L KH_2_PO_4_, 1.0 g/L (NH_4_)_2_SO_4_, 80 mg/L MgSO_4_·7H_2_O, 1 mg/L CaSO_4_·2H_2_O, 0.56 mg/L NiSO_4_·7H_2_O, 0.4 mg/L ferric citrate, and 200 mg/L NaHCO_3_.

For gas fermentation, cultures were diluted into 20 mL minimal medium in the serum bottle to an OD_600_ of ~ 0.2, and then a total 150 kPa of mixture gas [(O_2_:H_2_:CO_2_ = 10:80:10); Airkorea corporation, South Korea] was pressurized into the headspace of the serum bottle (20 mL of minimal medium in 157 mL of serum bottle) every 24 h. For the growth of engineered strain, 200 μg/mL kanamycin was included in the plates or media to maintain the pBBR1 plasmid. *Escherichia coli* DH10β employed for DNA manipulation was cultured at 37 °C in LB medium supplemented with 50 μg/mL kanamycin.

### Plasmid construction

All plasmids used in this study are listed in Table 1. The vector pBBR1MCS2 was a gift from Kenneth Peterson (Addgene plasmid #85168) [49]. The homologous genes (*regA* and *cbbR*) were amplified from the wild type *C. necator* H16 genome. The heterologous genes from *Synechocystis* sp. PCC6803 (*rbcL, rbcS, rbcX*) were codon-optimized according to the codon usage of *C. necator* H16 using Gene designer (Atum, California, USA) and synthesized by IDT KOREA. All designated primers used in this study are listed in Additional file 1: Table S2. All plasmid constructions were performed in *E. coli* DH10β and transformed into *C. necator* H16 using the electroporation method. Plasmid isolation and DNA purification were carried out using Mini exprep plasmid SV (Geneall, Korea) and QIAquick gel extraction kit (Qiagen, Germany), respectively.

### Quantification of PHB

Quantification of PHB was performed according to the modified method of Law and Slepecky (1961) [50]. The cultures were centrifuged for 10 min at 4200 rpm and washed twice with deionized water. Next, the pellets were frozen in – 80 °C and dried using a freeze dryer (Operon, Gimpo, Korea). After transferring to a 1.5 mL microcentrifuge tube, 0.5 mL of 95% H_2_SO_4_ (Junsei Chemical, Tokyo, Japan) was added to the pellet and vortexed. The tubes were incubated at 95 °C for 1 h. Finally, the samples were diluted to 20–50 times and analyzed by high pressure liquid chromatography (Agilent technology 1260 Infinity, CA, USA) with HIPLEX-H column (300 × 7.7 mm, Agilent technology, CA, USA) using UV/Vis detector and the 5 mM H_2_SO_4_ was used as mobile phase at 0.6 mL/min.

### RNA-sequencing analysis

For transcriptomic comparison of the control and engineered strains, RNA‐sequencing analyses were performed using tools from the commercial RNA‐Seq service Ebiogen, Inc. After 72 h of gas fermentation under nitrogen-limited conditions with the initial optical density of 2, the bacterial cells were harvested by centrifugation at 4200 rpm and 4 °C for 10 min. Total RNA was isolated with Trizol reagent (Invitrogen) according to the manufacturer's instructions. The purity and integrity of each total RNA sample were assessed according to the 28S/18S ratio and RNA integrity number measured on the 2100 Bioanalyzer system (Agilent Technologies). The cDNA library was generated using the Clontech SMARTer Stranded RNA‐Seq kit (Clontech). High‐throughput sequencing was performed on an Illumina HiSeq 2500 system (Illumina, Inc).

## Supplementary Information

**Additional file 1**: **Table S1.** Transcriptional changes of genes involved in metabolism of *cbbR* and *regA*-overexpressing strain. **Table S2.** Primers used in this work. **Figure S1.** PHB accumulation of the control and cbbR/regA overexpressed strains with the different initial optical densities of 0.2 and 2 under nitrogen-limited conditions (0.2 g/L of (NH_4_)_2_SO_4_) at 24 h and 168 h during the autotrophic culture. The gene of interest was induced by adding 0.2% (w/v) of l-arabinose at 24 h of the autotrophic culture. The *C. necator* H16 strain harboring pBAD empty vector was used as a control. The data represent the means of duplicate or triplicate experiments.

## Data Availability

All data for this study are included in this published article and its additional file.
