# Supplementary material for: Engineering Cupriavidus necator H16 for enhanced lithoautotrophic poly(3-hydroxybutyrate) production from CO2
Source: Microb Cell Fact. 2022 Nov 5;21:231. doi: 10.1186/s12934-022-01962-7 (PMC9636797; doi:10.1186/s12934-022-01962-7)
Supplement: Supplementary file 1 — Additional file 1: Table S1. Transcriptional changes of genes involved in metabolism of cbbR and regA-overexpressing strain. Table S2. Primers used in this work. Figure S1. PHB accumulation of the control and cbbR/regA overexpressed strains with the different initial optical densities of 0.2 and 2 under nitrogen-limited conditions (0.2 g/L of (NH4)2SO4) at 24 h and 168 h during the autotrophic culture. The gene of interest was induced by adding 0.2% (w/v) of l-arabinose at 24 h of the autotrophic culture. The C. necator H16 strain harboring pBAD empty vector was used as a control. The data represent the means of duplicate or triplicate experiments. [file 12934_2022_1962_MOESM1_ESM.docx]

**Additional file 1**

**Engineering *Cupriavidus necator* H16 for enhanced lithoautotrophic poly(3-hydroxybutyrate) production from CO_2_**

Soyoung Kim^1^, Yong Jae Jang^1^, Gyeongtaek Gong^1,2^, Sun-Mi Lee^1,2^, Youngsoon Um^1,2^, Kyoung Heon Kim^3^, Ja Kyong Ko^1,2,^*

^1^Clean Energy Research Center, Korea Institute of Science and Technology (KIST), Seoul, 02792, Republic of Korea

^2^Division of Energy and Environment Technology, KIST School, University of Science and Technology, Seoul 02792, Republic of Korea

^3^Department of Biotechnology, Graduate School, Korea University, Seoul 02841, Republic of Korea

* Correspondence to J.K. Ko: [jkko@kist.re.kr](mailto:jkko@kist.re.kr)

**Table S1.** Transcriptional changes of genes involved in metabolism of *cbbR* and *regA*-overexpressing strain

| Metabolism | Gene | Description | Fold change |
| --- | --- | --- | --- |
| CO_2_ transport | *can*  (H16_A0169) | Carbonic anhydrase; Interconversion between CO_2_ and carbonate | 1.8 |
| Protein folding | *groELb*  (H16_A1997) | plays an essential role in assisting protein folding (HSP60 family) | 2.7 |
|  | *groEL*  (H16_A0706) | plays an essential role in assisting protein folding | 1.7 |
| Flagella | *fliC*  (H16_B2360) | Flagellin (major structural component of flagella) | 0.19 |
|  | *flgE* (H16_B0264) | flagellar hook protein | 0.35 |
|  | *fliH*  (H16_B2370) | flagellar assembly protein | 0.34 |
|  | *flgN*  (H16_B0258) | flagellar biosynthesis protein | 0.36 |
|  | *flgI*  (H16_B0268) | flagellar basal body P-ring protein | 0.42 |
|  | *spoT2* (H16_A1337) | GTP pyrophosphokinase | 1.6 |
|  | *fabG* (H16_B0361) | 3-Oxoacyl-[acyl-carrier-protein] reductase | 35.6 |
|  | H16_A3311 | Enoyl-CoA hydratase (involved in PHB mobilization) | 0.43 |
|  | *paaH1* (H16_A0282) | 3-Hydroxyacyl-CoA dehydrogenase; catalyze conversion of acetoacetyl-CoA to the S-stereoisomer of 3-hydroxybutyryl-CoA | 0.63 |

**Table S2.** Primers used in this work

| Primers | Sequence |
| --- | --- |
| rbcLXS-saci | gagctcatggtgcaggccaaggccg |
| rbcLXS-hindiii | aagcttttagtagcggccctg |
| regA-f | aattcaaaagatcttttaagaaggaacaaccatgaccgacaccctcacc |
| regA-r | tcgaggtcgacggtatcgataagcttgcggcctttttctttaccgcg |
| cbbR-f | aattcaaaagatcttttaagaaggagagctcatgtcgtccttcctgcgcg |
| cbbR-r | cctgcagcccgggggatccactagttctagacagccaaccctcctggaacc |
| cbbR-regA-f | cgggtggcctgaggttccaggagggaccacaatgaccgacaccctcacc |


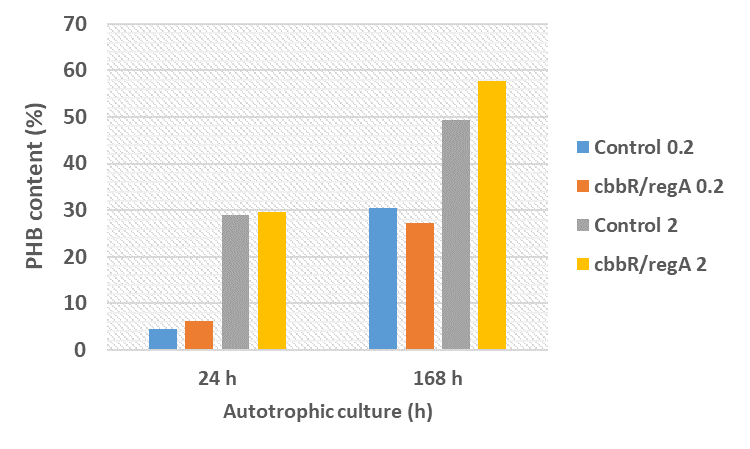


**Fig. S1.** PHB accumulation of the control and cbbR/regA overexpressed strains with the different initial optical densities of 0.2 and 2 under nitrogen-limited conditions (0.2 g/L of (NH_4_)_2_SO_4_) at 24 h and 168 h during the autotrophic culture. The gene of interest was induced by adding 0.2% (w/v) of L-arabinose at 24 hr of the autotrophic culture. The *C. necator* H16 strain harboring pBAD empty vector was used as a control. The data represent the means of duplicate or triplicate experiments.
